# Dysregulation of the FGF21–Adiponectin Axis in a Large Cohort of Patients with Severe Obesity and Liver Disease

**DOI:** 10.3390/ijms26178510

**Published:** 2025-09-02

**Authors:** Helena Castañé, Andrea Jiménez-Franco, Alina-Iuliana Onoiu, Vicente Cambra-Cortés, Anna Hernández-Aguilera, David Parada, Francesc Riu, Antonio Zorzano, Jordi Camps, Jorge Joven

**Affiliations:** 1 Unitat de Recerca Biomèdica, Hospital Universitari de Sant Joan, Institut d’Investigació Sanitària Pere Virgili, Universitat Rovira i Virgili, 43204 Reus, Spain; helena.castane-vilafranca@uni-tuebingen.de (H.C.); andrea.jimenez@urv.cat (A.J.-F.); alinaiuliana.onoiu@urv.cat (A.-I.O.); vicente.cambra@urv.cat (V.C.-C.); 2 Department of Pathology, Hospital Universitari de Sant Joan, Institut d’Investigació Sanitària Pere Virgili, Universitat Rovira i Virgili, 43204 Reus, Spain; anna.hernandez@salutsantjoan.cat (A.H.-A.); david.parada@salutsantjoan.cat (D.P.); francesc.riu@salutsantjoan.cat (F.R.); 3 Institute for Research in Biomedicine (IRB Barcelona), Department of Biochemistry and Molecular Medicine, Universitat de Barcelona, 08028 Barcelona, Spain; antonio.zorzano@irbbarcelona.org; 4 The Campus of International Excellence Southern Catalonia, 43003 Tarragona, Spain

**Keywords:** adipose tissue-liver axis, choline metabolism, interorgan crosstalk, MASLD, MASH, microbiota, organokines

## Abstract

We investigated the impact of liver damage on systemic inter-organ communication in an extensive observational case–control study of 923 patients with severe obesity and biopsy-confirmed metabolic dysfunction-associated steatotic liver disease (MASLD) or metabolic dysfunction-associated steatohepatitis (MASH) undergoing bariatric surgery. Using a comprehensive panel of circulating organokines, including fibroblast growth factor (FGF) 19, FGF21, adiponectin, galectin-3, irisin, and leptin, along with choline metabolites, we characterized metabolic signaling patterns associated with liver disease severity. Compared to controls, patients with MASLD/MASH exhibited significantly lower levels of FGF19, choline, and trimethylamine, while FGF21, galectin-3, irisin, and leptin were elevated. Sex-specific alterations in leptin and adiponectin were observed in patients with severe obesity but not in controls. Network analysis revealed a complex and individualized interplay among organokines, shaped by age, sex, and anthropometric factors. Despite this complexity, a dysregulation of the FGF21–adiponectin axis was associated with more advanced liver involvement. The large cohort and comprehensive organokine profiling studied provide valuable insights into the role of the FGF21–adiponectin axis on systemic metabolic alterations in severe obesity and their potential clinical implications.

## 1. Introduction

Different organisms have evolved sophisticated communication systems among their organs to coordinate metabolic responses efficiently in changing environmental and nutritional conditions. However, chronic nutrient excess and impaired nutrient processing can disrupt these inter-organ communication pathways, compromising metabolic homeostasis. This disruption has led to a growing public health challenge characterized by obesity and associated metabolic diseases, including liver dysfunction [1,2]. These metabolic abnormalities often coexist and may further interfere with organ crosstalk, especially in severe or morbid obesity, complicating personalized management strategies. Clinically, severe or morbid obesity is defined by a body mass index (BMI) of 40 kg/m^2^ or greater, or a BMI of 35 kg/m^2^ or greater with associated comorbidities. This condition frequently involves liver damage and is linked to an increased risk of serious metabolic complications [3,4].

Understanding the relationship between severe obesity and the progression of metabolic dysfunction-associated steatotic liver disease (MASLD) is critical. Patients advancing from MASLD to metabolic dysfunction-associated steatohepatitis (MASH) and fibrosis face a substantially heightened risk of cirrhosis and hepatocellular carcinoma [5]. Importantly, adipose tissue expansion beyond a certain threshold leads to metabolic dysregulation, highlighting the necessity for proactive interventions such as aggressive weight loss strategies and emerging pharmacological therapies [6,7].

Recent advances have reframed adipose tissue as an active endocrine organ coordinating systemic metabolism rather than merely an energy reservoir [8,9,10]. Communication among metabolic organs, including adipose tissue, liver, gut, and skeletal muscle, is primarily mediated by secreted factors known as organokines [11,12]. This “organokine crosstalk” is a complex network where hormones and metabolites function as signaling molecules that modulate energy homeostasis and tissue function [13,14]. For example, these signals can regulate liver fat accumulation and impact gut microbiota composition, bile acid metabolism, and lipid handling [15,16,17].

Key organokines such as fibroblast growth factors FGF19 and FGF21, adiponectin, galectin-3, irisin, and leptin represent well-characterized mediators of inter-organ communication with direct implications in obesity-related metabolic dysfunction. FGF19 and FGF21 are critical regulators of bile acid metabolism, glucose control, and lipid handling [18]; adiponectin exerts anti-inflammatory and insulin-sensitizing effects [19]; leptin is a key adipose-derived hormone modulating appetite and energy balance [20]; irisin is linked to muscle–adipose crosstalk and thermogenesis [21]; and galectin-3 has been implicated in fibrogenesis and metabolic processes [22].

However, the detailed molecular mechanisms governing these interactions remain incompletely understood, particularly in the context of severe obesity, due to a lack of studies combining large patient cohorts, biopsy-confirmed liver disease, and comprehensive investigation of circulating organokines and metabolic markers [23].

Treatment options for severe obesity are limited, with bariatric surgery being the only consistently effective intervention for sustained weight loss and metabolic improvement. However, not all patients are candidates, and long-term outcomes remain variable [24]. Understanding the metabolic alterations and disrupted inter-organ communication that accompany this disease is essential to identifying new biomarkers and therapeutic targets. Unfortunately, few studies have addressed these mechanisms in depth, especially in large cohorts with biopsy-proven liver disease and comprehensive organokine and metabolite profiling.

Building on previous work implicating mitochondrial dysfunction and organelle interplay in liver metabolic impairment [25,26], the present study aims to deepen the understanding of how organokine networks are altered across the spectrum of liver damage and metabolic dysfunction in an extensive cohort of patients with severe obesity. By systematically profiling key circulating organokines and related metabolites, we seek to elucidate the complex inter-organ communication patterns that characterize metabolic complications in severe obesity and explore their potential clinical implications.

## 2. Results

### 2.1. Participants

Anthropometric measurements and laboratory tests confirmed the link between excess body fat, metabolic dysfunction, and liver damage (Table 1). Patients with severe obesity shared a similar ethnic background and originated from the same geographical area. Sex distribution was noticeably skewed, with a significantly higher proportion of women than men. Patients showed a significantly higher prevalence of type 2 diabetes mellitus, hypertension, and dyslipidemia, which was reflected in elevated levels of glucose, insulin, triglycerides, and aminotransferases. In terms of organokines and choline metabolites, they showed lower concentrations of FGF19, choline, and trimethylamine (TMA), and higher concentrations of FGF21, irisin, and leptin. Analysis of liver histological features showed no significant differences based on sex. Therefore, we present data for the entire cohort (Supplementary Tables S1 and S2). The prevalence of MASLD was high, characterized by a complex distribution of liver lesions across a broad spectrum. Only 43 patients had a nonalcoholic fatty liver disease activity score (NAS) of 0, while 217 (24.2%) exhibited definite MASH (Supplementary Table S1 and Figure S1). Metabolic dysfunction correlated with the severity of liver damage. In patients with MASH, older age, higher anthropometric measurements, comorbidities, elevated plasma levels of FGF21 and lower adiponectin levels were evident (Supplementary Table S2).

No sex-related differences were observed in organokines and metabolites within the control group. However, among patients with severe obesity, plasma levels of leptin and adiponectin were higher in women than in men (Supplementary Figure S2).

### 2.2. Fibroblast Growth Factor 19 and Choline Metabolism

**Figure 1:**
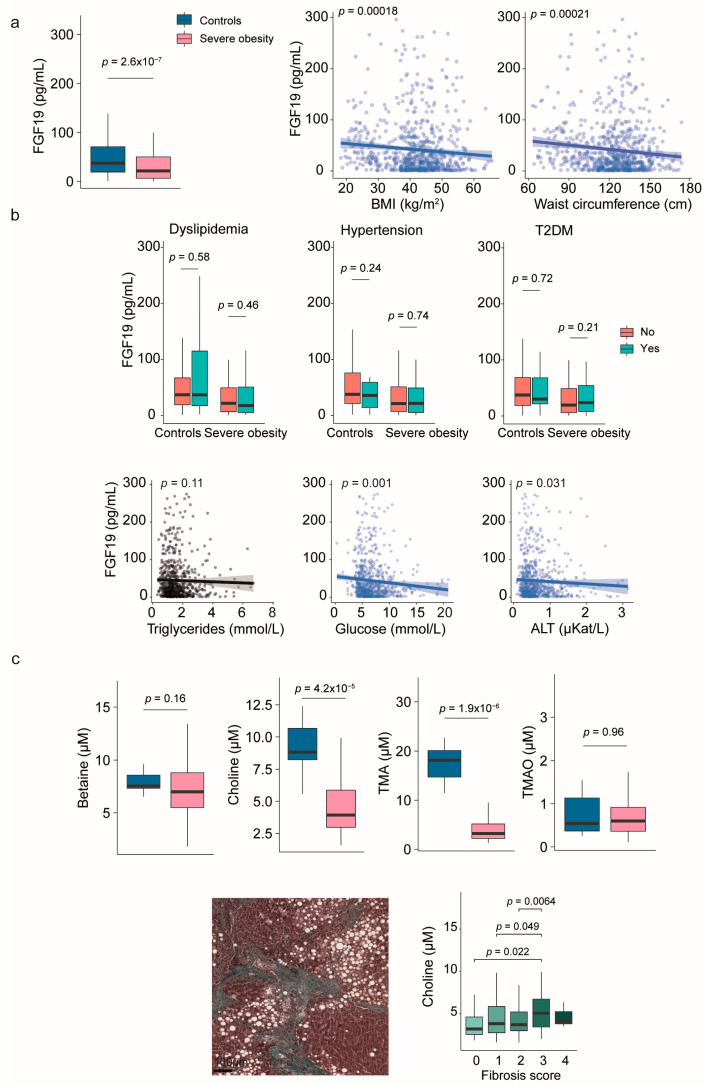
Evaluation of the gut-liver axis in severe obesity through fibroblast growth factor (FGF) 19 levels and choline metabolism markers. (**a**) Circulating levels of FGF19 were lower in individuals with severe obesity compared to controls and inversely correlated with body size. (**b**) There was no association between plasma FGF19 levels and metabolic comorbidities. (**c**) Choline and trimethylamine (TMA) concentrations were significantly lower in patients with obesity than in the control group, but progressively increased with fibrosis stages within the obese cohort. Representative histological images corresponding to the different fibrosis stages are provided in Supplementary Figure S1c. Statistical differences between groups were assessed using the Mann–Whitney U test. Correlations between variables were evaluated using Spearman’s rank correlation coefficient (Spearman’s ρ). ALT: Alanine aminotransferase; BMI: Body mass index; T2DM: Type 2 diabetes mellitus; TMAO: Trimethylamine N-oxide.

Patients with severe obesity had significantly lower circulating FGF19 levels than controls, which inversely correlated with body size measurements (Figure 1a). These differences were independent of dyslipidemia, hypertension, or diabetes (Figure 1b). Plasma FGF19 levels remained unaffected by varying degrees of liver damage (Supplementary Table S2). Specifically, there were no significant differences between patients with and without MASH, or associations with liver histological features (Supplementary Table S3).

As shown in Supplementary Figure S3, the small intestine is the primary source of FGF19, and circulating metabolites related to choline metabolism can influence communication between the gut and the liver. Our study found that plasma levels of choline and TMA were significantly lower in individuals with severe obesity compared to the control group, and these levels were not associated with body measurements. The differences in betaine and trimethilamine N-oxide (TMAO) levels did not reach statistical significance (Figure 1c), nor did we observe any relationships with metabolic comorbidities. However, ordinal logistic regression analyses revealed significant associations with hepatic histological features. Specifically, plasma choline levels increased with the NAS and were associated with a higher fibrosis score. Plasma levels of choline and TMAO were higher in patients with the highest ballooning scores (Figure 1c and Supplementary Table S3).

### 2.3. Fibroblast Growth Factor 21

**Figure 2:**
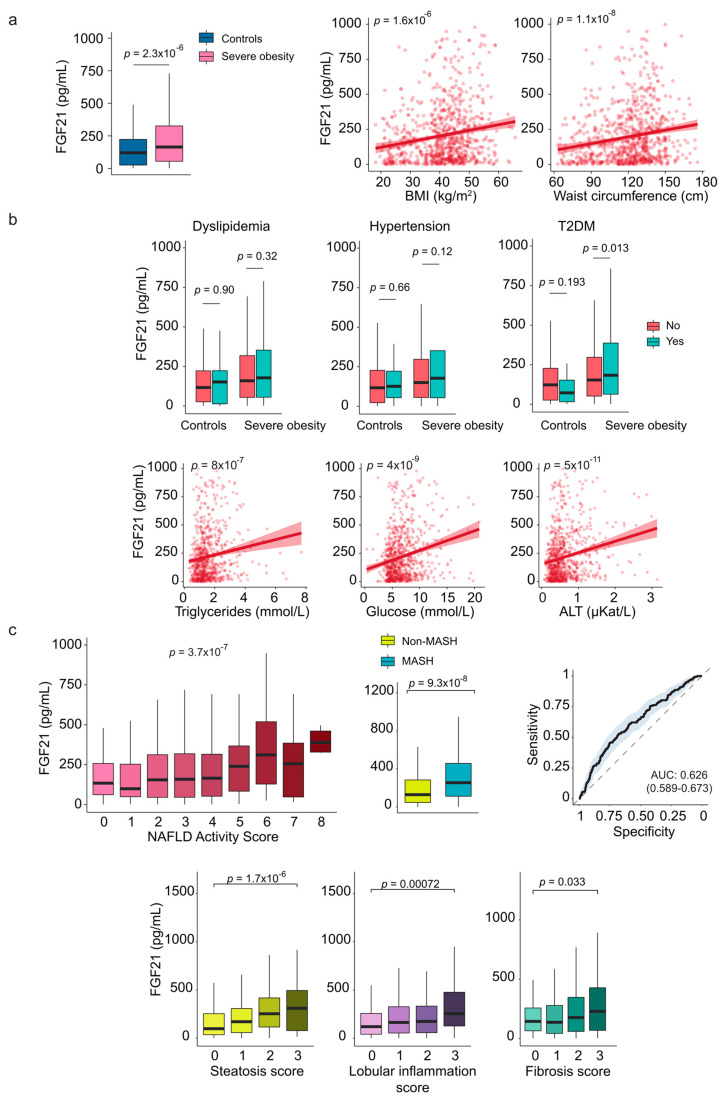
The association of circulating fibroblast growth factor (FGF) 21 with hepatic histological features in severe obesity. (**a**) Circulating levels of FGF21 were higher in individuals with severe obesity than in the control group and showed a positive correlation with body size. (**b**) Box plots illustrate the association between FGF21 levels and diabetes. Scatter plots show the relationship with laboratory markers. (**c**) Box plots demonstrate the relationship between FGF21 levels, hepatic histological features, and predictive indicators of metabolic dysfunction-associated steatohepatitits (MASH). The receiver operating characteristic plot indicates that FGF21 is not an effective marker for distinguishing between patients with and without MASH. Representative histological images for each group are shown in Supplementary Figure S1c. Statistical differences between groups were assessed using the Mann–Whitney U or the Kruskal–Wallis tests. Correlations between variables were evaluated using Spearman’s rank correlation coefficient (Spearman’s ρ). ALT: Alanine aminotransferase; AUC: Area under the curve; T2DM: Type 2 diabetes mellitus.

Patients with severe obesity had significantly higher levels of circulating FGF21 than the control group. These elevated levels positively correlated with various measures of body size (Figure 2a). Plasma FGF21 levels were also associated with common markers of liver injury and glucose metabolism. Although we did not find any significant associations with dyslipidemia or hypertension, it is noteworthy that patients with severe obesity and diabetes had higher FGF21 concentrations than those without diabetes (Figure 2b).

The liver is the primary source of FGF21, and circulating FGF21 levels closely correlate with liver histopathology. Patients with MASH exhibited significantly higher plasma FGF21 concentrations than those without MASH. Furthermore, there were robust correlations between FGF21 levels and the NAS, with positive relationships for lobular inflammation, steatosis, and fibrosis. Receiver operating characteristic curve analyses indicated that FGF21 has limited accuracy as a non-invasive diagnostic marker. Nonetheless, its significant associations with metabolic comorbidities and liver pathology suggest potential utility in guiding therapeutic strategies (Figure 2c and Supplementary Tables S2 and S3).

### 2.4. Galectin-3

Galectin-3, previously known as macrophage antigen 2 (Mac-2), is a multifunctional protein produced and secreted by various types of cells. In our study cohort, we found that plasma levels of galectin-3 were positively correlated with BMI and were significantly higher in patients with severe obesity compared to controls (Supplementary Figure S4a). Additionally, patients with diabetes had markedly elevated levels of galectin-3 in their circulation, with no associated changes related to hypertension or dyslipidemia (Supplementary Figure S4b). MASH did not affect plasma galectin-3 levels. However, we did find that plasma galectin-3 decreased in patients with greater liver fibrosis. Furthermore, galectin-3 was consistently associated with liver macrophages (Supplementary Figure S4c and Tables S2 and S3).

### 2.5. Irisin

The muscle generates the highest amounts of irisin. We observed that patients with severe obesity had higher circulating irisin levels than controls. However, plasma irisin levels showed a poor correlation with anthropometric measurements (Supplementary Figure S5a). In contrast to controls, the presence of dyslipidemia and hypertension was significantly associated with lower circulating levels of irisin in patients with severe obesity. There was no significant relationship between irisin levels and diabetes (Supplementary Figure S5b). Although we found a positive correlation with the ballooning score, our results do not indicate strong associations with MASH in these patients (Supplementary Figure S5c and Tables S2 and S3).

### 2.6. Leptin and Adiponectin: The Relevance of Sex Dimorphism

**Figure 3:**
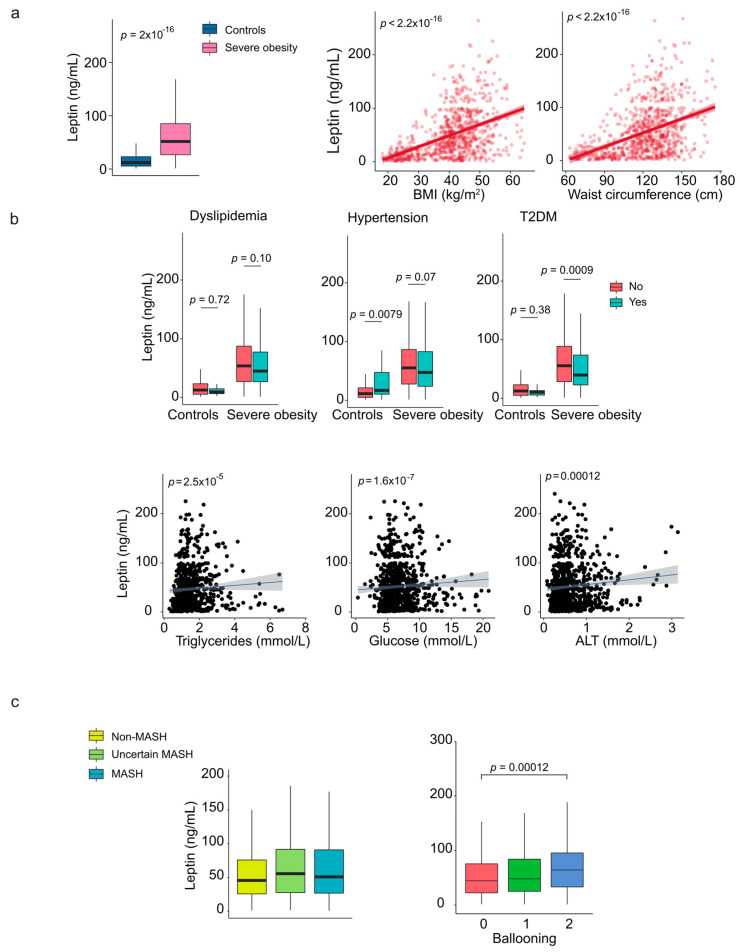
The relationship between circulating leptin and comorbidities. (**a**) Patients with severe obesity exhibited higher circulating levels of leptin compared to controls, which positively correlated with anthropometric measurements. (**b**) Boxplots and scatter plots show the association between leptin levels and laboratory markers. (**c**) Leptin levels were associated with ballooning score. Representative histological images for each ballooning score are shown in Supplementary Figure S1c. Statistical differences between groups were assessed using the Mann–Whitney U or the Kruskal–Wallis tests. Correlations between variables were evaluated using Spearman’s rank correlation coefficient (Spearman’s ρ). ALT: Alanine aminotransferase; BMI: Body mass index; MASH: Metabolic dysfunction-associated steatohepatitis; T2DM: Type 2 diabetes mellitus.

When analyzing the entire cohort, we found that circulating leptin levels were higher in patients with severe obesity than in controls, and there was a significant positive correlation between anthropometric measures and leptin (Figure 3a). However, plasma leptin levels were lower in patients with metabolic comorbidities, with a statistically significant trend noted only among those with diabetes (Figure 3b). Liver damage did not appear to influence the levels of this adipokine, and we found no appreciable differences between patients with and without MASH (Supplementary Table S2). Interestingly, patients with more ballooned liver cells had significantly higher plasma leptin concentrations than those without ballooning (Figure 3c, Supplementary Table S3).

We also noted significant sex differences in our findings. Women with severe obesity had higher circulating leptin levels than men (Supplementary Figure S2) and these levels strongly correlated with body size measurements for both sexes when BMI was considered as a confounding factor (Supplementary Figure S6a). Notably, the impact of diabetes varied by sex; it was not significant in men, while women with diabetes had lower plasma leptin levels compared to those without diabetes (Supplementary Figure S6b). The pattern was not observed in the control group. Furthermore, the relationship between circulating leptin and ballooning score was significant only in women (Supplementary Figure S6c, Table S4).

**Figure 4:**
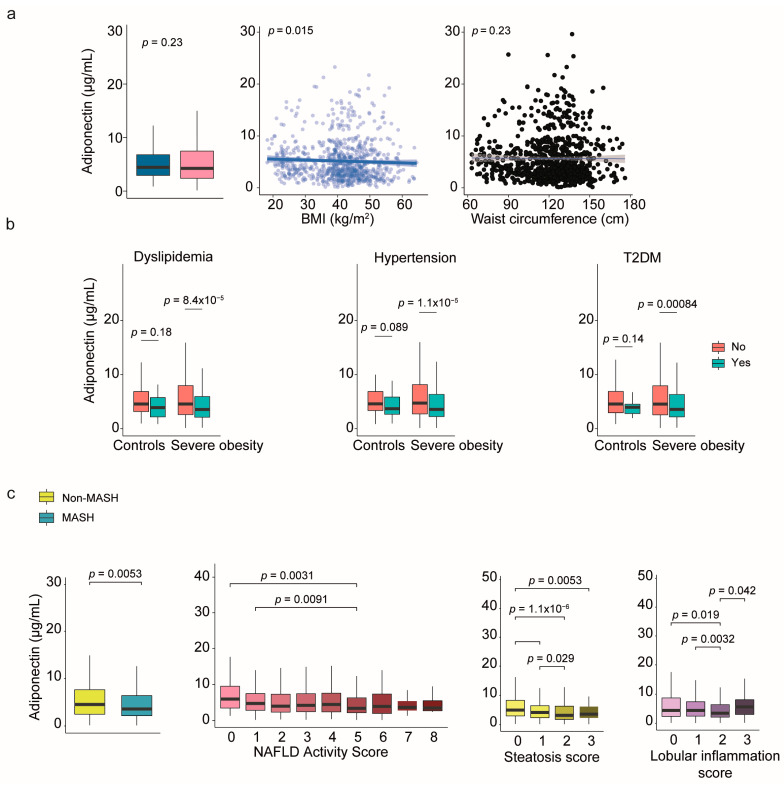
The relationship between circulating adiponectin and comorbidities. (**a**) Plasma adiponectin levels were generally lower in patients with more severe conditions and showed little correlation with body size. (**b**) In contrast to healthy controls, low adiponectin levels were associated with dyslipidemia, hypertension, and diabetes. (**c**) Reduced adiponectin levels may serve as a marker to differentiate patients with MASH from those without. Representative histological images steatosis and lobular inflammation scores are shown in Supplementary Figure S1c. Statistical differences between groups were assessed using the Mann–Whitney U or the Kruskal–Wallis tests. Correlations between variables were evaluated using Spearman’s rank correlation coefficient (Spearman’s ρ). BMI: Body mass index; MASH: Metabolic dysfunction-associated steatohepatitis; NAFLD: Nonalcoholic fatty liver disease; T2DM: Type 2 diabetes mellitus.

In contrast to the findings related to leptin, when analyzing the entire cohort, plasma adiponectin levels did not show significant differences between patients with severe obesity and the control group. Additionally, the correlation between adiponectin levels and body size measurements was weak (Figure 4a). However, circulating adiponectin levels were lower in men compared to women (Supplementary Figures S2 and S7a). When the data was separated by sex, accounting for BMI as a potential confounder, there were no notable changes in the association with morphometric values (Supplementary Figure S7a). On the other side, metabolic comorbidities significantly affected patients with obesity; we observed that lower plasma adiponectin levels were associated with dyslipidemia, hypertension, and diabetes, regardless of sex (Figure 4b and Supplementary Figure S7b). Furthermore, liver damage had a significant impact, and lower plasma adiponectin levels could modestly predict the presence of MASH in patients (Supplementary Table S2). We also found significant associations between plasma adiponectin levels and steatosis, inflammation, and NAS (Figure 4c and Supplementary Table S3) with only minor differences observed between the sexes (Supplementary Figure S7c, Table S4).

### 2.7. Network Modeling and Insights into Patterns: The Functional Overlap Between FGF21 and Adiponectin

Mixed graphical models are advanced statistical tools that allow researchers to visualize and understand complex relationships between different types of variables simultaneously. In this study, we used these models to map the intricate connections between organokines, liver damage markers, and metabolic complications in patients with severe obesity. The goal was to identify patterns or clusters of co-regulated molecules that might serve as diagnostic tools or reveal underlying biological mechanisms.

**Figure 5:**
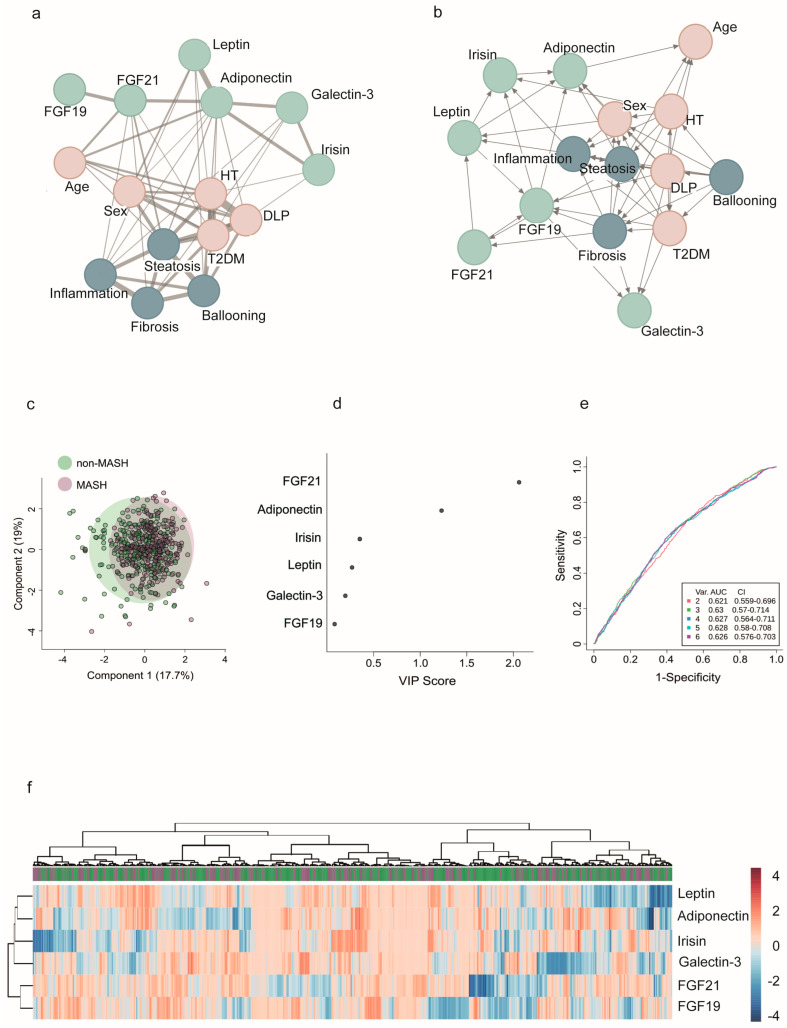
Modeling the complexity of communication among organokines and clinical variables. (**a**) Mixed graphical models represent a network of clinical and biochemical variables, where nodes correspond to variables, and edges indicate significant positive or negative correlations or interactions. (**b**) Probabilistic inferences Bayesian network highlight the conditional dependencies and inferred directionality among variables. (**c**) Principal component analysis did not reveal different patterns between patients with and without metabolic dysfunction-associated steatohepatitits (MASH). (**d**) The variable importance plot shows that the fibroblast growth factor (FGF) 21 and adiponectin were relevant factors in assessing MASH. (**e**) All classifier models demonstrated similar performance in receiver operating characteristic curves, with low areas under the curve (AUC). (**f**) However, analysis does not identify any potential clusters of co-regulated organokines.

In patients with severe obesity, we often encounter varied data modalities. We used mixed graphical models to investigate the relationships between different organokines, liver damage, and other metabolic comorbidities. This approach provides a flexible framework for representing conditional dependencies (Figure 5a,b). Network analyses revealed that all organokines are interconnected, and clinical factors and histological features can complicate the interpretation of their interactions. Multivariate statistical techniques and regression models did not identify specific clusters of co-regulated organokines, and adjusted models did not clarify their potential use in diagnostics.

The disruption in communication is most noticeable in the context of liver damage. For example, the collective evaluation of organokines was unable to differentiate between MASH and non-MASH patients. The degree of liver damage has varying effects on circulating signals, indicating distinct metabolic profiles (Figure 5c–f). The principal component analysis did not effectively separate the groups, and the heatmap illustrates the complex clustering of patients based on circulating organokine levels. Similarly, the receiver operating characteristic curves show only slight variations in the already modest performance of models that include clinical data and organokine levels. However, the plot depicting variable importance in projection scores highlights the significant roles of FGF-21 and adiponectin in distinguishing the effects of liver lesions (Figure 5d).

**Figure 6:**
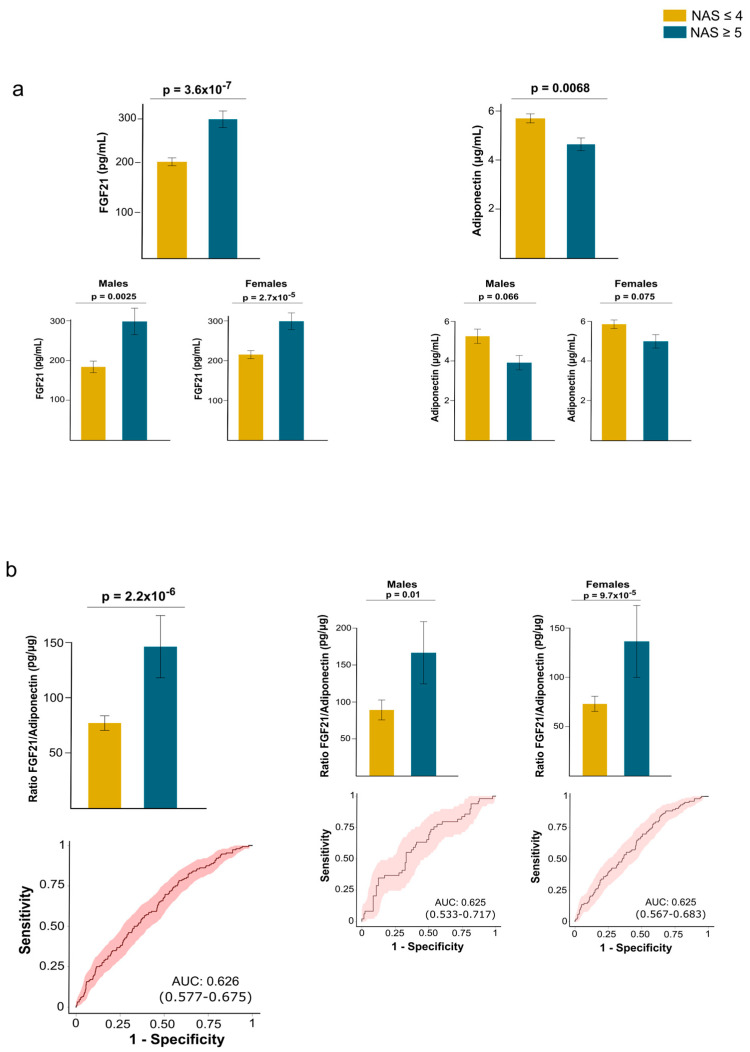
Circulating fibroblast growth factor (FGF21) and adiponectin levels in patients with and without metabolic dysfunction-associated steatohepatitits (MASH). (**a**) In patients with MASH, circulating FGF21 levels are increased, whereas plasma adiponectin concentrations are reduced. Sex influences results obtained for adiponectin. (**b**) This inconsistency disappeared when calculating the ratio between both hormones, which provided modest predictive value for distinguishing patients with MASH, as calculated by receiver operating characteristics curves. Statistical differences between groups were assessed using the Mann–Whitney U test. AUC: Area under the curve; NAS: Nonalcoholic fatty liver disease activity score.

This finding is significant because FGF21 and adiponectin exhibit functional similarities despite differences in their plasma concentrations. Our data suggest that an impaired FGF21–adiponectin axis may be crucial to developing MASH. In patients with MASH, circulating FGF21 levels were elevated, while plasma adiponectin concentrations were decreased compared to patients with a less severe form of MASLD. Additionally, we observed sex-based differences in these levels. The results regarding circulating FGF21 levels were consistent for both men and women; however, while there was a similar trend for circulating adiponectin levels, the differences did not reach statistical significance in either males or females (Figure 6a). To clarify this relationship, we calculated the FGF21/adiponectin ratio and observed that patients with MASH had elevated values compared to those without it. These results were consistent across both males and females and demonstrated a modest predictive value for liver damage (Figure 6b).

## 3. Discussion

Understanding the complex interplay between severe obesity, metabolic dysfunction, and liver disease is essential for developing effective diagnostic and therapeutic strategies. We examined patients with severe obesity and biopsy-proven liver disease, integrating clinical, histological, and biochemical data to analyze circulating organokines and choline-related metabolites. This approach aimed to identify key signaling alterations that reflect or drive hepatic and metabolic changes.

Severe obesity disrupts the homeostatic balance between major metabolic organs, including the liver, adipose tissue, skeletal muscle, and gut, partly through impaired organokine communication [6,10,27,28]. Our findings demonstrate significant alterations in circulating levels of FGF19, FGF21, leptin, irisin, and adiponectin, providing evidence that severe obesity is associated with dysregulated organokine networks that contribute to liver disease development and progression.

The liver serves as the primary line of defense against gut-derived substances, positioning it at the center of the interplay between intestinal signals and systemic metabolism. In severe obesity, excessive adiposity alters this gut-liver axis, potentially disrupting hepatocyte-mediated regulation of FGF19, an enterokine produced in the ileum in response to bile acid signaling [15,16,17,18,19,20,21,22,23,29,30]. We found decreased plasma FGF19 levels in patients with severe obesity, consistent with downregulation of the farnesoid X receptor (FXR) in the gut, a key regulator of FGF19 expression. Given FXR’s role in bile acid homeostasis and metabolic signaling, these observations support exploring therapeutic strategies targeting FXR pathways, including peroxisome proliferator-activated receptors and FGF19-based analogs [31,32,33].

The observed decrease in FGF19 levels was not directly associated with comorbidities. However, metabolic signals from the gut encompass more than protein hormones alone. Our results on choline and TMAO metabolism offer additional insight into gut-liver crosstalk in severe obesity. Despite known literature variability [34], our data reveal significant reductions in circulating choline and TMA levels compared to controls, with both correlating with liver fibrosis scores. Interestingly, plasma TMAO levels remained stable, potentially reflecting compensatory hepatic responses involving FGF19-regulated pathways, such as bile acid signaling and one-carbon metabolism [35]. Although FGF19 plays a well-established role in bile acid homeostasis, we observed no significant correlation between plasma FGF19 levels and liver injury severity. This apparent paradox suggests that circulating FGF19 reflects intestinal FXR activity and gut-derived metabolic signals rather than hepatic damage severity [31,32,33]. In severe obesity, alterations in enterohepatic signaling or fibroblast growth factor receptor 4 responsiveness may occur independently of histological liver injury [36,37]. These data support the concept that low FGF19 levels characterize severe obesity but do not necessarily indicate fibrosis severity.

Most circulating FGF21 is produced in the liver and affects other tissues through specific receptors [38,39]. In contrast to FGF19, plasma FGF21 concentrations were elevated in patients with severe obesity, especially in those with diabetes, and were strongly associated with liver damage. These elevated FGF21 levels in MASH patients likely reflect a compensatory response to metabolic stress, underscoring its role as a key regulator in conditions of hepatic and systemic dysfunction. Recent clinical trials have examined FGF21 analogs such as aldafermin and pegozafermin, which reduce hepatic fat in MASH treatment but show variable patient responses [40,41]. Non-responders may include individuals with FGF21 resistance or altered hepatic FGF21 expression [42]. Our data indicate that such expression differences relate closely to liver damage severity in severe obesity and may contribute to the modest diagnostic accuracy for MASH observed in our cohort [23,43,44,45]. The FGF21 to adiponectin ratio improves interpretation.

Galectin-3, a β-galactose-binding lectin, is widely recognized as a key mediator of inflammation and fibrogenesis and has been proposed as a prognostic biomarker for hepatocellular carcinoma [46,47,48]. In our study, plasma galectin-3 levels were elevated in patients with severe obesity compared to controls but showed an unexpected inverse correlation with liver fibrosis scores, contrasting with its established pro-fibrotic role. One possible explanation is that galectin-3 levels are stage-dependent: while they contribute to fibrogenesis in early disease, they may plateau or decline in advanced stages, when tissue remodeling slows. The concomitant increase in FGF21 and decrease in FGF19 suggest a shift in hepatic stress and bile acid signaling, as FGF21 may exert anti-inflammatory effects that suppress galectin-3 expression [38,39]. Additionally, chronic obesity may alter fibroblast or macrophage function, limiting galectin-3 production. These mechanisms may collectively explain the lower galectin-3 levels observed despite the progression of fibrosis. This interpretation, however, remains speculative. We confirmed galectin-3 expression in hepatic macrophages, supporting its involvement in obesity-related metabolic inflammation. Ongoing clinical trials with Selvigaltin, a galectin-3 inhibitor, may provide further insight into galectin-3’s role in MASH pathogenesis and progression [49,50].

Circulating irisin, a myokine associated with muscle size and activity, was elevated in patients with severe obesity compared to controls. This finding is consistent with earlier research on overweight or obese individuals but somewhat surprising given that these patients generally engage in low levels of physical activity [51]. Like other organokines, irisin promotes energy expenditure by facilitating non-shivering thermogenesis [52]. Patients with low plasma irisin levels demonstrated significant metabolic dysregulation, particularly dyslipidemia and hypertension, suggesting a protective regulatory role in severe obesity. Although irisin’s contribution to liver pathology remains unclear, we observed a positive correlation between irisin levels and hepatocyte ballooning, despite no correlation with the NAS [43,53].

Sex differences significantly influence disease progression and treatment responses in severe obesity. Fat distribution differs between sexes and links to metabolic health, with men generally showing greater susceptibility to metabolic comorbidities and more pronounced clinical responses to weight loss [54,55]. In our cohort, we observed sexual dimorphism in the circulating adipokines leptin and adiponectin. Plasma leptin levels were significantly elevated in patients with severe obesity and correlated closely with body mass index and waist circumference. These elevated leptin levels support the concept of leptin resistance, which contributes to chronic positive energy balance [56,57]. While leptin is a reliable marker of body fat, its role in liver pathology is not appreciated. Despite the importance of communication between adipose tissue and the liver [6], leptin did not correlate with MASLD or MASH in our cohort.

This study found no significant differences in circulating adiponectin levels between patients with severe obesity and controls. However, plasma adiponectin concentrations were lower in men than women, with weak correlations to body size. Low adiponectin levels were significantly associated with metabolic dysfunction and were reduced in patients with MASH, consistent with adiponectin’s protective role against liver steatosis and fibrosis [14,58,59,60]. This inverse relationship between adiponectin levels and liver disease severity emphasizes adiponectin’s protective potential and suggests that reduced levels may contribute to MASH progression. Therapeutically, interventions to increase adiponectin levels could potentially slow or prevent liver disease progression in obese individuals [61,62,63]. We identified a notable contrast between FGF21 and adiponectin: while adiponectin levels were reduced in MASH patients, FGF21 levels were increased. This imbalance in the FGF21-adiponectin axis may be crucial for liver health, though liver disease itself may also contribute to this dysregulation (Supplementary Figure S8).

Network analysis visualizes the complexity of organokine communication and conditional dependencies associated with metabolic abnormalities and liver damage. However, multivariate analysis and regression models did not reveal clusters of co-regulated organokines or predictive models distinguishing patients with and without MASH. The adipose tissue-liver axis remains critical for restoring liver homeostasis, with weight loss playing a central role [43,44,63,64,65]. Recent MASH treatments include weight loss-dependent peptide-based incretin therapies targeting glucagon-like peptide-1 and glucose-dependent insulinotropic polypeptides, as well as weight loss-independent approaches such as FGF21 analogs (which increase adiponectin levels) and thyroid hormone receptor beta agonists like Resmetirom [66,67,68]. However, clinical trials have not clarified the role of inter-organ communication, and drugs effective for liver disease often inadequately address concurrent metabolic comorbidities, and vice versa [69]. Our findings demonstrate relevant factors impacting organokine expression and function. While organokine interconnectedness exists, this relationship remains unclear in severe obesity, potentially explaining why most medications show inconsistent therapeutic effects.

## 4. Strenghts and Limitations of the Study

This study provides a comprehensive analysis of circulating organokines and choline metabolites in a well-characterized cohort of patients with severe obesity undergoing bariatric surgery, with liver histology available for most cases. The simultaneous assessment of multiple signaling pathways allowed us to explore their associations with both metabolic traits and liver histological features. However, cross-sectional design limits causal interpretation, and circulating levels may not fully reflect tissue-specific activity or receptor responsiveness. Additionally, our study cohort included predominantly female participants, reflecting the typical profile of patients undergoing bariatric surgery. This sex imbalance limits the generalizability of our findings and highlights the need to further explore sex-specific differences in organokine signaling and disease progression in future studies. Moreover, findings may not be generalizable to other populations, as lifestyle, genetic background, and microbiome composition can influence these molecular patterns. Consequently, some secondary findings should be interpreted as exploratory and warrant confirmation in independent cohorts.

## 5. Materials and Methods

### 5.1. Study Design

We conducted an observational case–control study involving 923 consecutive patients with severe obesity and biopsy-proven MASLD from the EOM cohort (ClinicalTrials.org reference number NCT05554224). All eligible patients met the inclusion criteria for bariatric surgery at the Hospital Universitari de Sant Joan in Reus, Spain. These criteria required participants to be over 18 years old and to have a BMI ≥ 35 kg/m^2^. All patients underwent laparoscopic sleeve gastrectomy, during which liver biopsy samples were systematically obtained for histological assessment. We excluded individuals with clinical or analytical evidence of severe illness, chronic or acute inflammation, cancer or infectious diseases. Patients showing histological patterns of hepatocellular damage suggestive of alcoholic disease were also excluded. The reference group comprised 258 non-obese individuals. Ultrasonography and laboratory analyses suggested the absence of liver disease. All were matched for age and ethnic background. These participants were drawn from a population study and follow-up conducted by the Institut d’Investigació Sanitària Pere Virgili in our area by the procedures outlined in the Declaration of Helsinki [70]. The study received approval from our institution’s ethics committee (EPIMET PI21/00510_083, PL4NASH112/2021 and EOM 244/2024), and all participants provided written informed consent.

### 5.2. Sampling and Data Collection

We collected blood samples from all participants between 8:00 and 9:00 A.M., ensuring a 10 h fasting period beforehand. Plasma was obtained by collecting blood in EDTA tubes. The samples were processed within two hours and plasma were stored at −80 °C until analysis. No control was applied for menstrual cycle, menopause, or hormone use in women. We followed conventional protocols and laboratory methods to obtain anthropometric measurements and to diagnose type 2 diabetes mellitus, hypertension, and dyslipidemia in all participants [10]. In patients, we examined liver biopsies obtained during surgery. We conducted laboratory analyses and ultrasonography for the control group to confirm the likely absence of significant liver disease.

### 5.3. Biochemical and Histological Assessments

We obtained four-micron sections from liver tissues fixed in formalin and embedded in paraffin. We assessed steatosis, ballooning, and lobular inflammation on hematoxylin and eosin-stained sections following the validated NAS [43,71,72]. A re-review protocol determined that 27 biopsies lacked sufficient quality and were excluded from the analysis. As a result, histological analysis was conducted on 896 biopsies by a single experienced pathologist blinded to clinical data. Using a final score ranging between 0 and 8, we identified 340 patients with NAS < 3 (non-MASH), 339 with NAS 3-4 (uncertain MASH), and 217 with NAS of 5 or greater (definite MASH). Fibrosis staging was performed on Masson’s trichrome-stained sections, and in some cases, we used the SAF algorithm (steatosis, activity and fibrosis) for reassessment [10]. This categorization provides insights into the different manifestations of liver conditions. When necessary, we used immunochemistry as described [30]. We analyzed serum levels of commonly measured analytes, including glucose, insulin, triglycerides, total cholesterol, high-density lipoprotein cholesterol, low-density lipoprotein cholesterol, and aminotransferases, using a COBAS^®^ 8000 automatic analyzer (Roche Diagnostics, Basel, Switzerland). Additionally, we measured the plasma concentrations of a selected panel of organokines that previously showed responses to nutritional states in an exploratory study [30]. This panel included FGF19, FGF21, galectin-3, leptin, adiponectin, and irisin, which were analyzed using human-validated enzyme-linked immunosorbent assay (ELISA) kits from R&D Systems (Minneapolis, MN, USA) with the following catalog numbers: #DY969, #DY2539, #DY1154, #DY398, #DY1065, and #DY9420-05, respectively. All assays were performed according to the manufacturer’s instructions, with appropriate controls for inter-assay variability. Each sample was analyzed once without repetition.

### 5.4. Mass Spectrometry for Selected Metabolites

We included the measurement of betaine, choline, TMA, and TMAO concentrations using liquid chromatography coupled to triple quadrupole mass spectrometry. Their association with organokines and their role in the metabolic crosstalk between the liver, gut, and adipose tissue has been described [34]. Briefly, 100 μL of plasma were mixed with 900 μL of methanol: water (8:2) and ten μL of internal standard (betaine-D9, choline-D9, TMAO-D9 at ten ppm each). Afterwards, the mix was incubated at −20 °C for 2 h to precipitate proteins. Samples were centrifuged at 14,000× *g* rpm for 10 min at 4 °C, and the supernatants were mixed with 75 μL of 50 mM tert-butyl bromoacetate in acetonitrile and ten μL of 70% ammonium hydroxide. Samples were vortexed for 1 min and incubated for 30 min at room temperature. We added 50 μL of 1% formic acid in acetonitrile, and samples were vortexed and centrifuged again at 14,000 rpm for 5 min at 4 °C. Supernatants were collected into chromatographic vials for subsequent analysis. Samples (two μL) were injected into an Ultra-High-Performance Liquid Chromatography (UHPLC) 1290 Infinity II Series instrument (Agilent, Santa Clara, CA, USA), coupled to a triple quadrupole equipped with an electrospray ionization source QqQ/MS 6470 Series (Agilent) operating in positive-ion mode. The chromatographic column for the UHPLC was an ACQUITY UPLC BEH HILIC 1.7 μm, 2.1 × 100 mm (Waters, Milford, MA, USA). The mobile phase consisted of A: acetonitrile: water (1:9) and B: acetonitrile: water (9:1) in 10 mM ammonium formate and 0.125% formic acid. The gradient consisted of 0% A for 1 min, 10% A at 4 min, and 55% for 5 min at a constant flow rate of 0.5 mL/minute. Instrument conditions included a nebulizer gas pressure of 50 psi, a gas temperature of 300 °C, a gas flow rate of 11 L/min, a sheath gas temperature of 400 °C, a sheath gas flow rate of 12 L/min, a capillary voltage of 2500 V, and a nozzle voltage of 500 V. Each metabolite was quantified by correcting the peak areas with their corresponding internal standard and calibration curve using the MassHunter Workstation Quantitative Analysis B.10.0 software (Agilent). All relevant analytical information has been previously described [73,74].

### 5.5. Statistical Analyses

Statistical analyses were performed using RStudio (R version 4.0.2). The Shapiro–Wilk normality test was used to assess the normality of the variables. Since some variables did not present normal distributions, non-parametric methods were used to ensure consistency. Two- and multi-group comparisons were performed using the Mann–Whitney U and Kruskal–Wallis tests. For categorical comparisons, we used the Fisher Exact test. Appropriate corrections for multiple comparisons were applied where indicated to control for Type I and Type II errors. The “Readxl” and “dplyr” packages were used for data management in R. Descriptive statistics, including participants’ characteristics, were generated with the “Tableone” package, with continuous variables summarized as medians and interquartile ranges and categorical variables as counts and percentages. Graphical resources were created using the “ggplot2”, “ggpur”, and “pROC” packages to obtain box and bar plots, correlation plots, and receiver operating characteristic curves. For ordinal logistic regression, we utilized the “ordinal” package. We used the R “mgm package” to implement and visualize conditional relationships between both continuous and categorical variables within a unified network structure. The model included regularization to ensure robustness, and the resulting networks highlight relevant inter-variable dependencies. The analysis was performed following the guidelines and recommendations provided in the official package documentation (https://cran.r-project.org/web/packages/mgm/mgm.pdf, accessed on 13 March 2025). Finally, MetaboAnalyst 6 was used to obtain hierarchically clustered heatmaps and to run Partial Least Square Discriminant Analysis.

## 6. Conclusions

Providing personalized care for patients with severe obesity remains a challenge due to the heterogeneous response to treatment, although weight loss remains the most effective intervention. Our findings highlight the role of inter-organ crosstalk in the development of MASLD and other metabolic complications in these patients. We identified disruptions in organokine signaling, particularly involving adipose tissue and the liver. FGF21 and adiponectin emerged as key mediators, showing interconnected patterns and similar associations with metabolic health and liver disease. These results suggest that therapeutic strategies aimed at restoring the FGF21–adiponectin axis, together with weight reduction, may enhance the management of MASLD. Longitudinal studies are needed to clarify the temporal dynamics of this axis, but these findings offer a foundation for transforming MASLD treatment through targeted organokine-based interventions in patients with severe obesity, though the need for sequential liver biopsies remains a significant limitation for future research.

## Figures and Tables

**Table 1:** Demographic and clinical characteristics of participants.

Variable	Controls (*n* = 258)	Severe Obesity (*n* = 923)	*p*-Value
Women, n (%)	121 (47.1)	677 (73.4)	<0.001
Age (years)	45 (35–62)	49 (41–56)	0.347
BMI (kg/m^2^)	26.7 (23.3–29.8)	43.9 (40.3–48.4)	<0.001
Waist circumference (cm)	89 (78–98)	130 (120–139)	<0.001
T2DM, n (%)	18 (7.0)	253 (27.4)	<0.001
Hypertension, n (%)	40 (15.5)	406 (44.0)	<0.001
Dyslipidemia, n (%)	25 (9.7)	234 (25.4)	<0.001
*Conventional biochemical variables*			
Glucose (mmol/L)	4.7 (4.3–5.2)	6.7 (5.5–8.5)	<0.001
Insulin (pmol/L)	47.0 (29.3–65.8)	67.8 (37.7–109.5)	<0.001
HOMA-IR	1.4 (0.9–2.2)	3.2 (1.7–5.7)	<0.001
Triglycerides (mmol/L)	1.0 (0.7–1.5)	1.5 (1.2–2.0)	<0.001
Cholesterol (mmol/L)	5.2 (4.7–5.9)	4.0 (3.5–4.7)	<0.001
LDL (mmol/L)	3.1 (2.6–3.8)	2.4 (1.9–3.0)	<0.001
HDL (mmol/L)	1.4 (1.2–1.8)	1.0 (0.8–1.2)	<0.001
ALT (μKat/L)	0.3 (0.2–0.4)	0.6 (0.4–0.9)	<0.001
AST (μKat/L)	0.3 (0.3–0.4)	0.6 (0.4–0.8)	<0.001
GGT (μKat/L)	0.2 (0.2–0.4)	0.4 (0.2–0.6)	<0.001
*Organokines and metabolites*			
FGF19 (pg/mL)	37.2 (19.0–70.7)	21.3 (6.2–49.9)	<0.001
Betaine (μM)	7.5 (7.3–8.6)	7.0 (5.5–8.8)	0.163
Choline (μM)	8.8 (8.2–10.7)	3.9 (3.0–5.9)	<0.001
TMA (μM)	18.1 (14.7–20.1)	3.2 (2.2–5.2)	<0.001
TMAO (μM)	0.5 (0.4–1.1)	0.6 (0.4–0.9)	0.957
FGF21 (pg/mL)	119.7 (25.6–222.8)	164.2 (54.5–326.1)	<0.001
Galectin-3 (ng/mL)	11.3 (6.2–17.3)	12.7 (6.6–21.2)	0.080
Irisin (ng/mL)	1.1 (0.6–1.6)	1.5 (0.8–2.4)	<0.001
Leptin (ng/mL)	12.0 (5.4–23.0)	51.6 (26.7–85.1)	<0.001
Adiponectin (μg/mL)	4.4 (2.9–6.8)	4.2 (2.4–7.5)	0.225

ALT: alanine aminotransferase; AST: aspartate aminotransferase; FGF: fibroblast growth factor; GGT: gamma-glutamyl transferase; HDL: high-density lipoprotein cholesterol; HOMA-IR: homeostatic model assessment for insulin resistance; LDL: low-density lipoprotein cholesterol; MASH: metabolic dysfunction-associated steatohepatitis; T2DM: type 2 diabetes mellitus; TMA: trimethylamine; TMAO: trimethylamine N-oxide.

## Data Availability

The datasets generated during and/or analyzed during the current study are available from the corresponding authors on reasonable request.

## Supplementary Materials

The following supporting information can be downloaded at: https://www.mdpi.com/article/10.3390/ijms26178510/s1.

## Abbreviations

The following abbreviations are used in this manuscript:

BMI	Body mass index
ELISA	Enzyme-linked immunosorbent assay
FGF	Fibroblast growth factor
FXR	Farnesoid X receptor
MASLD	Metabolic dysfunction-associated steatotic liver disease
MASH	Metabolic dysfunction-associated steatohepatitis
NAS	Nonalcoholic fatty liver disease activity score
TMA	Trimethylamine
TMAO	Trimethylamine N-oxide
UHPLC	Ultra-High-Performance Liquid Chromatography
