# Supplementary material for: Dysregulation of the FGF21–Adiponectin Axis in a Large Cohort of Patients with Severe Obesity and Liver Disease
Source: Int J Mol Sci. 2025 Sep 2;26(17):8510. doi: 10.3390/ijms26178510 (PMC12429463; doi:10.3390/ijms26178510)
Supplement: Supplementary file 1 [file ijms-26-08510-s001.zip › ijms-3800766-supplementary-Figure S8.pdf]

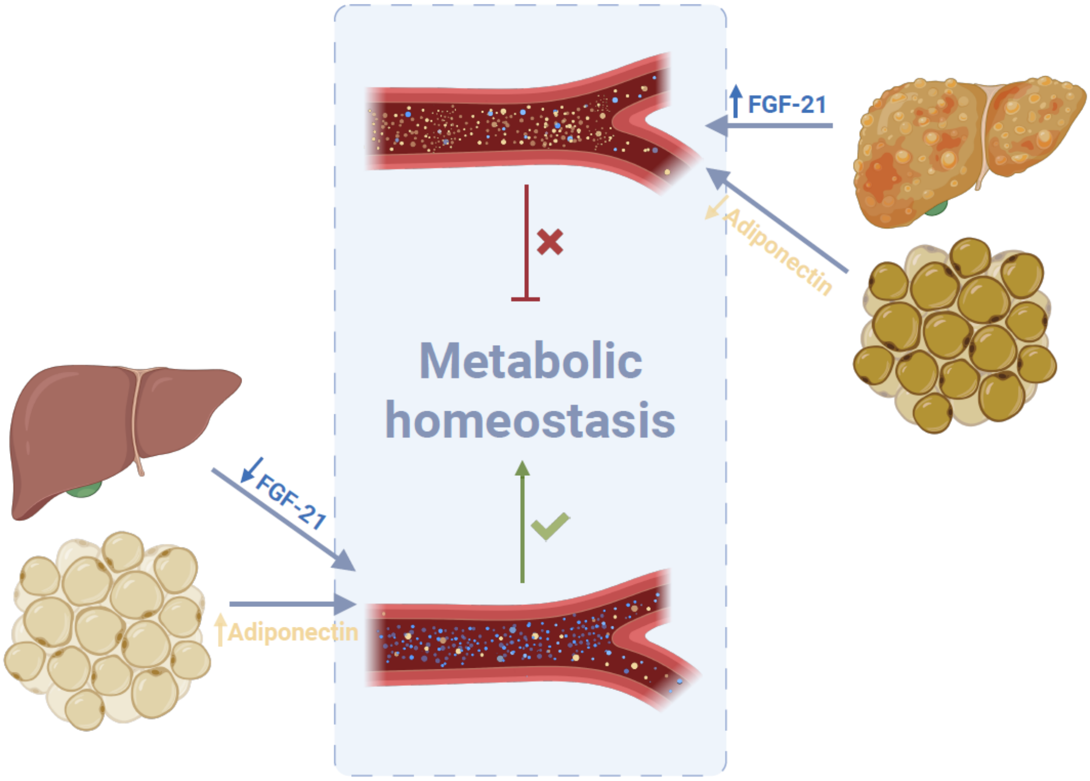

Supplementary Fig. S8. FGF21-adiponectin axis imbalance in severe obesity and liver disease. Schematic representation showing elevated circulating FGF21 levels and reduced circulating adiponectin levels in patients with severe obesity and MASH. This organokine imbalance disrupts metabolic homeostasis, while balanced signaling is required for normal metabolic function. The dysregulated FGF21-adiponectin axis may be both a consequence and contributor to liver disease progression.
