# Supplementary material for: Dysregulation of the FGF21–Adiponectin Axis in a Large Cohort of Patients with Severe Obesity and Liver Disease
Source: Int J Mol Sci. 2025 Sep 2;26(17):8510. doi: 10.3390/ijms26178510 (PMC12429463; doi:10.3390/ijms26178510)
Supplement: Supplementary file 1 [file ijms-26-08510-s001.zip › ijms-3800766-supplementary-Tables and Figures 1-7.pdf]

Supplementary information corresponding to the article:

Dysregulation of the FGF21–adiponectin axis in a large cohort of patients with severe obesity and liver disease

Helena Castañé[1], Andrea Jiménez-Franco[1], Alina-Iuliana Onoiu[1], Vicente Cambra-Cortés[1], Anna Hernández-Aguilera[2], David Parada[2], Francesc Riu[2], Antonio Zorzano[3], Jordi Camps[1,*], Jorge Joven[1,4*]

[1] Unitat de Recerca Biomèdica, Hospital Universitari de Sant Joan, Institut d'Investigació Sanitària Pere Virgili, Universitat Rovira i Virgili, 43204 Reus, Spain
[2] Department of Pathology, Hospital Universitari de Sant Joan, Institut d'Investigació Sanitària Pere Virgili, Universitat Rovira i Virgili, 43204 Reus, Spain
[3] Department of Biochemistry and Molecular Medicine, Universitat de Barcelona. Institute for Research in Biomedicine (IRB Barcelona), The Barcelona Institute of Science and Technology, Barcelona, Spain
[4] The Campus of International Excellence Southern Catalonia, 43003 Tarragona, Spain

* Correspondence: jorge.camps@salutsantjoan.cat (J. C.), jorge.joven@salutsantjoan.cat (J. J.).

Supplementary Table S1. Histological features of liver biopsies of patients with severe obesity.

	Non-MASH (*N* = 340)	Uncertain (*N* = 339)	MASH (*N* = 217)	*P* value
Steatosis score, n (%)				
<5%	252 (74.1)	77 (22.7)	-	
5-33%	84 (24.7)	196 (57.8)	34 (15.7)	
34-66%	4 (1.2)	62 (18.3)	113 (52.1)	<0.001
>66%	-	4 (1.2)	70 (32.3)	
Lobular inflammation score, n (%)				
None	97 (28.9)	15 (4.5)	-	
<2 foci	208 (61.9)	200 (59.3)	65 (30.0)	
2-4 foci	31 (9.2)	104 (30.9)	119 (54.8)	<0.001
>4 foci	-	18 (5.3)	33 (15.2)	
Ballooning score, n (%)				
None	231 (68.8)	73 (21.6)	5 (2.3)	
Few	95 (28.3)	158 (46.7)	85 (39.2)	<0.001
Many	10 (3.0)	107 (31.7)	127 (58.5)	
Fibrosis score, n (%)				
None	57 (16.9)	12 (3.6)	9 (4.3)	
Perisinusoidal or periportal	143 (42.4)	114 (34.2)	42 (20.1)	
Perisinusoidal and periportal	115 (34.1)	155 (46.5)	91 (43.5)	<0.001
Bridging	22 (6.5)	51 (15.3)	64 (30.6)	
Cirrhosis	-	1 (0.3)	3 (1.4)	
NAS histological scoring system, n (%)				
0	43 (12.6)	-	-	
1	118 (34.7)	-	-	
2	178 (52.4)	-	-	
3	1 (0.3)	190 (56.0)	-	<0.001
4	-	149 (44.0)	-	
5	-	-	120 (55.3)	
6	-	-	72 (33.2)	
7	-	-	21 (9.7)	
8	-	-	4 (1.8)	

MASH: metabolic dysfunction-associated steatohepatitis.

Supplementary Table S2. Clinical and laboratory characteristics according to liver damage distribution.

	Non-MASH (*N* = 340)	Uncertain (*N* = 339)	MASH (*N* = 217)	P value
Women, n (%)	260 (76.5)	247 (73.1)	151 (69.6)	0.194
Age (years)	47 (40 - 56)	49 (42 - 56)	51 (43 - 57)	0.038
BMI (kg/m^2)	43.4 (39.3 - 47.5)	44.1 (40.9 - 48.6)	44.7 (40.5 - 48.8)	0.030
Waist circumference (cm)	127 (117 - 136)	129 (121 - 139)	134 (123 - 142)	<0.001
T2DM, n (%)	66 (19.4)	96 (28.3)	82 (37.8)	<0.001
Hypertension, n (%)	125 (36.8)	150 (44.2)	118 (54.4)	<0.001
Dyslipidemia, n (%)	67 (19.7)	81 (23.9)	79 (36.4)	<0.001
Conventional biochemical variables				
Glucose (mmol/L)	6.4 (5.3 - 8.0)	6.9 (5.7 - 8.7)	7.1 (5.7 - 9.3)	0.001
Insulin (pmol/L)	63.2 (33.9 - 101.4)	66.9 (37.5 - 108.7)	75.7 (45.8 - 121.3)	0.011
HOMA-IR	2.7 (1.5 - 4.5)	3.3 (1.8 - 6.3)	3.7 (2.1 - 6.9)	0.001
TG (mmol/L)	1.4 (1.1 - 1.8)	1.6 (1.2 - 2.2)	1.7 (1.3 - 2.3)	<0.001
Cholesterol (mmol/L)	4.0 (3.4 - 4.7)	4.1 (3.6 - 4.8)	4.0 (3.5 - 4.8)	0.193
LDL (mmol/L)	2.4 (1.9 - 2.9)	2.5 (1.9 - 3.1)	2.4 (1.9 - 3.1)	0.743
HDL (mmol/L)	1.0 (0.9 - 1.3)	1.0 (0.8 - 1.2)	1.0 (0.8 - 1.2)	0.078
ALT (µKat/L)	0.5 (0.4 - 0.7)	0.6 (0.4 - 0.9)	0.8 (0.5 - 1.3)	<0.001
AST (µKat/L)	0.5 (0.4 - 0.7)	0.6 (0.4 - 0.8)	0.7 (0.5 - 1.0)	<0.001
GGT (µKat/L)	0.3 (0.2 - 0.5)	0.4 (0.2 - 0.5)	0.5 (0.4 - 0.7)	<0.001
Organokines and metabolites				
FGF19 (pg/mL)	21.5 (7.7 - 54.9)	21.7 (5.5 - 52.1)	21.3 (6.2 - 45.0)	0.755
Betaine (µM)	6.9 (5.5 - 9.1)	6.9 (5.5 - 9.2)	7.2 (5.4 - 8.2)	0.813
Choline (µM)	3.5 (2.7 - 5.6)	4.1 (3.1 - 5.7)	3.9 (3.1 - 6.7)	0.148
TMA (µM)	3.1 (2.2 - 5.0)	3.2 (2.3 - 4.9)	3.3 (2.1 - 5.8)	0.95
TMAO (µM)	0.6 (0.4 - 0.8)	0.6 (0.4 - 1.0)	0.6 (0.4 - 1.0)	0.682
FGF21 (pg/mL)	129.3 (47.0 - 284.5)	164.2 (50.3 - 318.3)	255.3 (112.7 - 458.7)	<0.001
Galectin-3 (ng/mL)	12.7 (6.5 - 19.8)	13.5 (7.2 - 21.7)	11.7 (5.7 - 21.6)	0.698
Irisin (ng/mL)	1.4 (0.7 - 2.2)	1.6 (0.8 - 2.5)	1.6 (0.9 - 2.4)	0.131
Leptin (ng/mL)	46.2 (26.0 - 77.4)	55.7 (27.8 - 92.2)	52.3 (27.0 - 91.4)	0.169
Adiponectin (µg/mL)	4.5 (2.5 - 7.7)	4.3 (2.4 - 7.5)	3.6 (2.2 - 6.4)	0.018
Ratio FGF21/Adiponectin (pg/µg)	21.6 (8.6 – 81.3)	33.0 (9.8 – 82.9)	55.5 (20.6 – 151.7)	<0.001

ALT: alanine aminotransferase; AST: aspartate aminotransferase; FGF: fibroblast growth factor; GGT: gamma-glutamyl transferase; HDL: high-density lipoprotein cholesterol; HOMA-IR: homeostatic model assessment for insulin resistance; LDL: low-density lipoprotein cholesterol; MASH: metabolic dysfunction-associated steatohepatitis; T2DM: type 2 diabetes mellitus; TG: triglycerides; TMA: trimethylamine; TMAO: trimethylamine N-oxide.

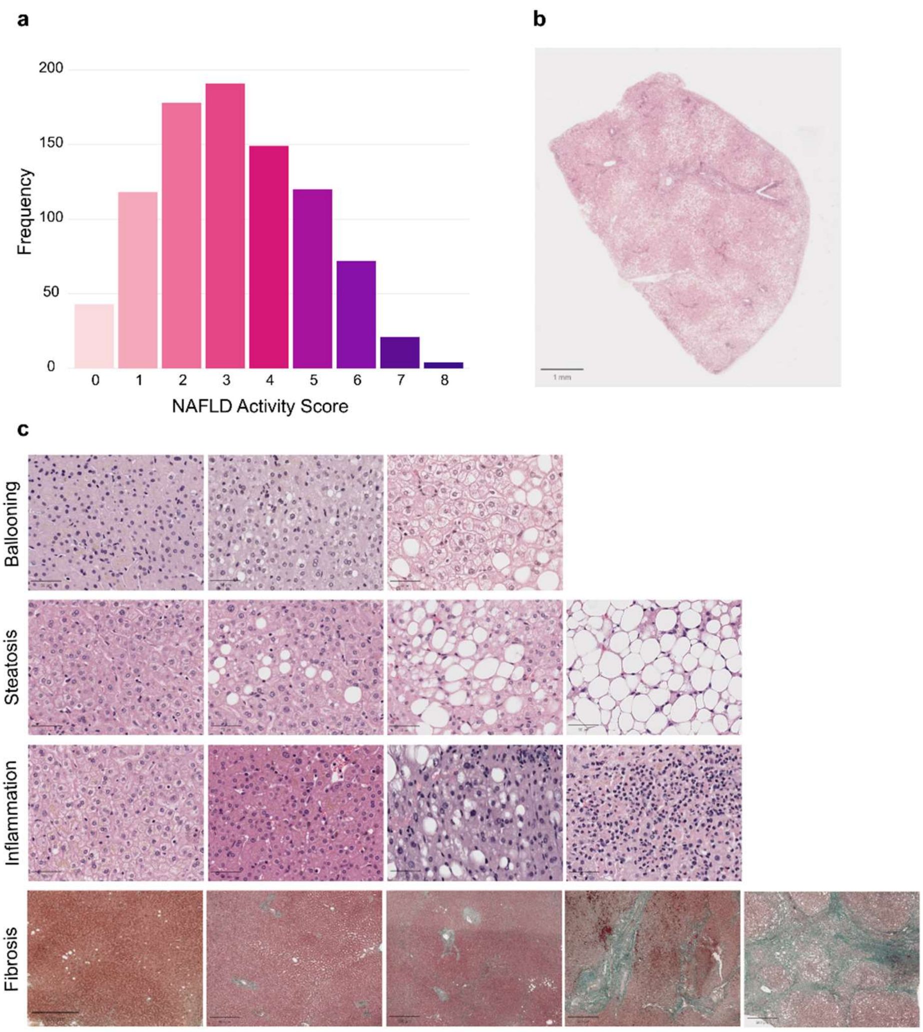

Supplementary Fig. S1. Histopathologic diagnosis of metabolic dysfunction-associated steatohepatitis (MASH) in patients with severe obesity. (a) Distribution of the Nonalcoholic Fatty Liver Disease (NAFLD) Activity Score (NAS) in our cohort. For clarity, we defined a score ≥ 5 as a surrogate marker for MASH; however, a score ≤ 4 did not necessarily indicate a benign histology. (b) We utilized whole slide images to assess relevant histological features. (c) Representative images illustrating the different categories of ballooning, steatosis, inflammation, and fibrosis, as defined by the NAS histological scoring system and detailed in Tables S1 and S2.

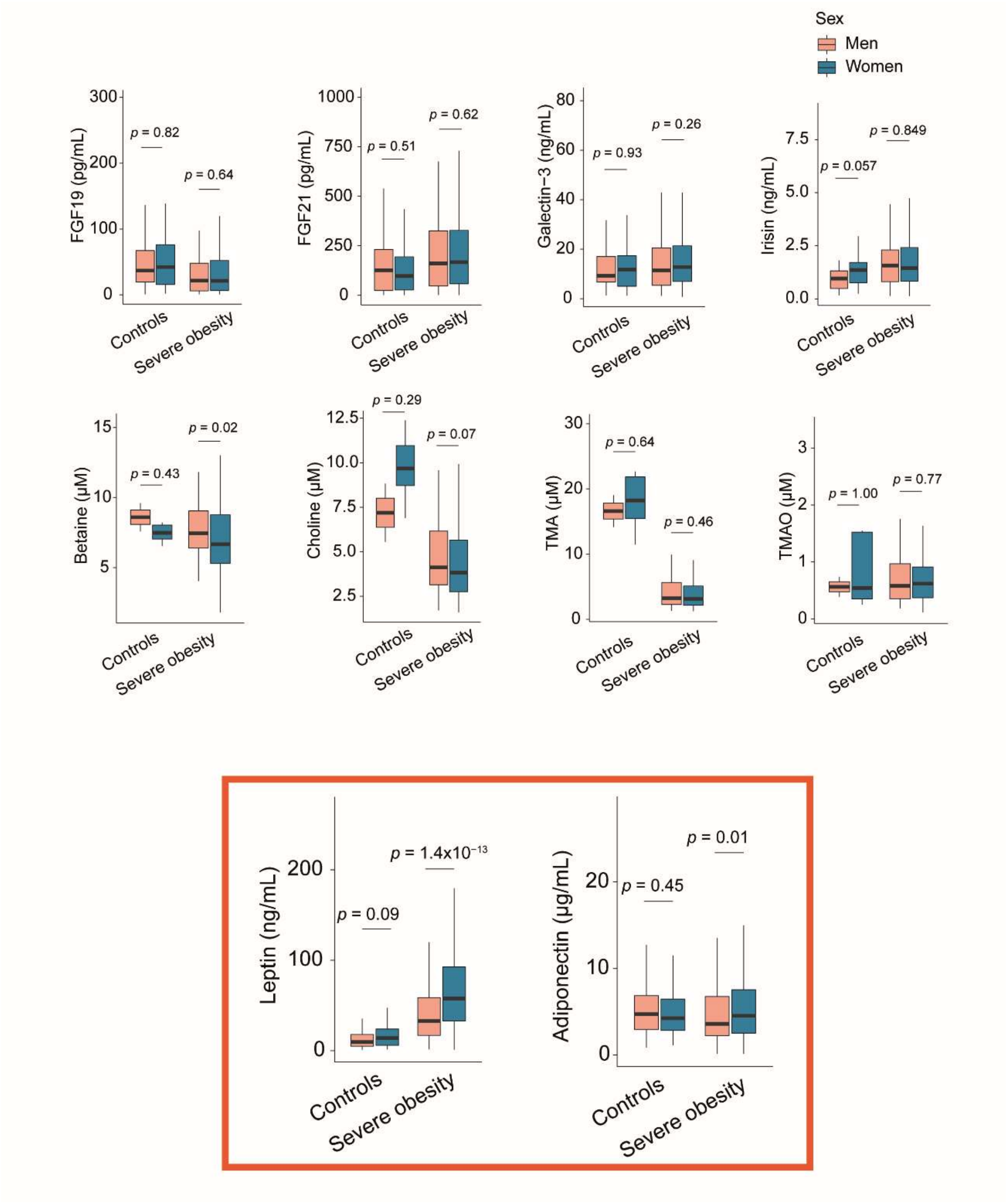

Supplementary Fig. S2. Box-and-whisker plots illustrate the variability outside the upper and lower quartiles for the measured variables. Notable sex differences were observed only in plasma levels of leptin and adiponectin in patients with severe obesity.

Statistical differences between groups were assessed using the Mann–Whitney U test. FGF: Fibroblast growth factor; TMA: Trimethylamine; TMAO: Trimethylamine N-oxide.

Supplementary Table S3. Ordinal logistic regression analysis summarizing the associations between organokines and metabolites with histopathology.

	Estimate	*P* value
NAS histological scoring system		
FGF19	-0.001	0.50
Betaine	-0.010	0.81
Choline	0.130	0.03
TMA	-0.006	0.90
TMAO	0.350	0.16
FGF21	0.001	3.7×10^{-7}
Galectin-3	-0.001	0.91
Irisin	0.070	0.22
Leptin	0.002	0.13
Adiponectin	-0.040	0.01
Steatosis score		
FGF19	-0.001	0.32
Betaine	-0.050	0.30
Choline	0.080	0.16
TMA	-0.070	0.23
TMAO	0.120	0.65
FGF21	0.002	2.6×10^{-11}
Galectin-3	0.003	0.65
Irisin	0.040	0.47
Leptin	0.001	0.74
Adiponectin	-0.080	1.3×10^{-6}
Lobular inflammation score		
FGF19	0.001	0.62
Betaine	0.005	0.91
Choline	0.100	0.11
TMA	0.030	0.60
TMAO	0.250	0.29
FGF21	0.001	0.01
Galectin-3	0.007	0.26
Irisin	-0.050	0.41
Leptin	-0.001	0.49
Adiponectin	-0.050	0.01
Ballooning score		
FGF19	-0.002	0.17
Betaine	0.020	0.75
Choline	0.130	0.04
TMA	0.070	0.22
TMAO	0.550	0.03
FGF21	0.001	0.31
Galectin-3	-0.010	0.03
Irisin	0.130	0.02
Leptin	0.005	0.01
Adiponectin	0.020	0.26
Fibrosis score		
FGF19	-0.001	0.34
Betaine	0.060	0.25
Choline	0.160	0.01
TMA	0.020	0.67
TMAO	-0.120	0.60
FGF21	0.001	0.01
Galectin-3	-0.010	0.01
Irisin	0.090	0.12
Leptin	-0.001	0.51
Adiponectin	-0.002	0.92

FGF: Fibroblast growth factor; NAFLD: non-alcoholic fatty liver disease, TMA: trimethylamine, TMAO: trimethylamine N-oxide. The column "estimate" represents the estimated coefficients (β) for each predictor in the model. These values indicate the effect of each independent variable on the probability that the ordinal dependent variable takes higher values on its scale. If $\beta > 0$, increasing the predictor increases the probability that the response falls into a higher category. If $\beta < 0$, increasing the predictor decreases the probability of being in a higher category. If $\beta \approx 0$, the predictor has little to no effect.

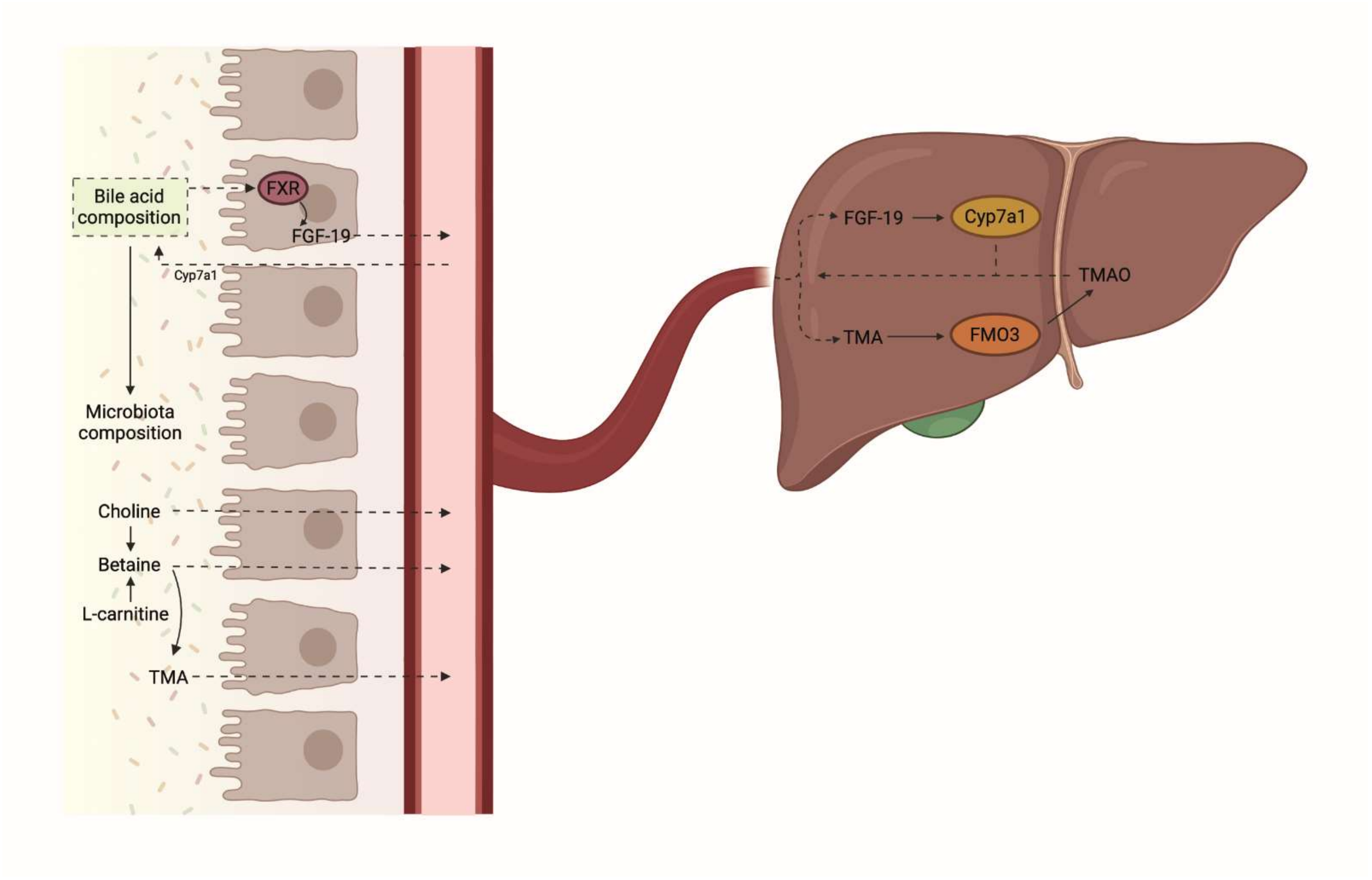

Supplementary Fig. S3. Schematic representation of the gut-liver axis, illustrating the potential relationships among the fibroblast growth factor (FGF19), bile acids, microbiota composition, and choline metabolism.
Cyp7a1: Cholesterol 7 alpha-hydroxylase; FMO3: Flavin-containing monooxygenase 3; FXR: Farnesoid X receptor; TMA: Trimethylamine; TMAO: Trimethylamine N-oxide.

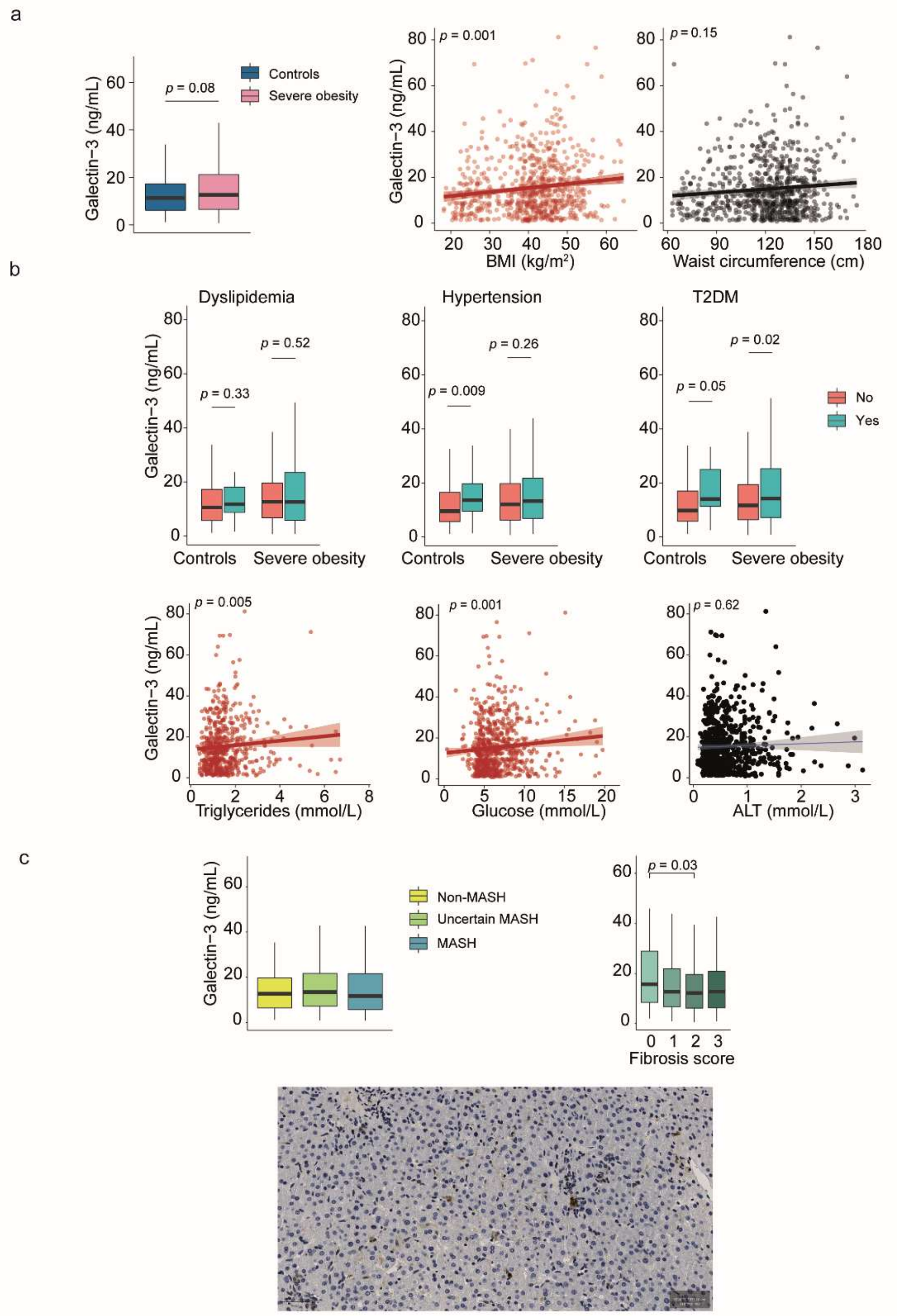

Supplementary Fig. S4. The relationship between circulating galectin-3 and comorbidities. (a) Circulating levels of galectin-3 in controls and patients with severe obesity correlated poorly with anthropometric measurements. (b) Boxplots and scatter plots show the association between galectin-3 levels and laboratory markers. Higher levels were associated with diabetes (T2DM). (c) Galectin-3 levels were associated fibrosis scores. Immunohistochemistry confirmed its expression in macrophages.

Statistical differences between groups were assessed using the Mann–Whitney U or the Kruskal-Wallis tests. Correlations between variables were evaluated using Spearman's rank correlation coefficient (Spearman's ρ). ALT: Alanine aminotransferase; BMI: Body mass index; MASH: Metabolic dysfunction-associated steatohepatitis.

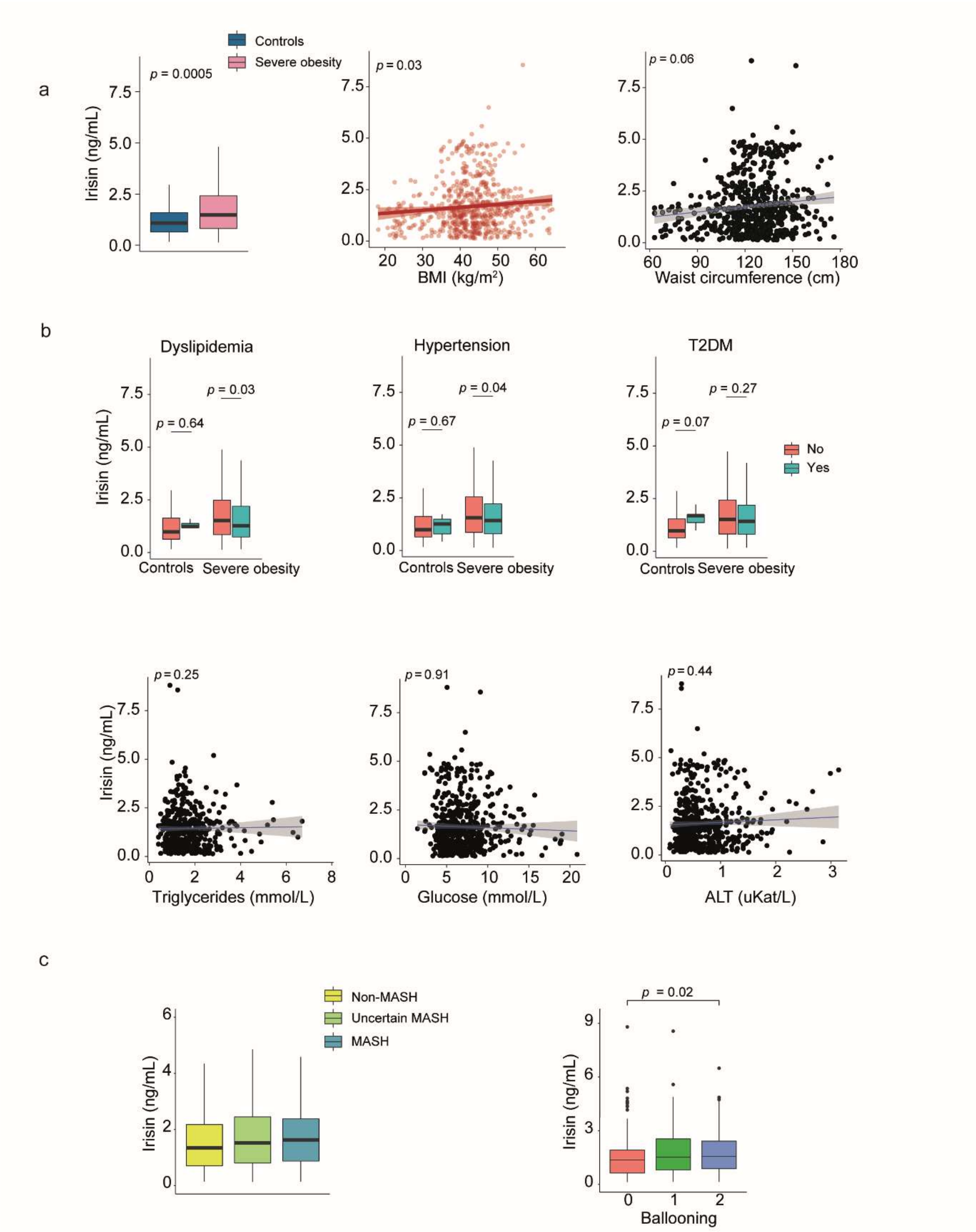

Supplementary Fig. S5. The relationship between circulating irisin and comorbidities. (a) Circulating irisin levels were higher in patients with severe obesity compared to controls, and they showed a poor correlation with anthropometric measurements. (b) Boxplots and scatter plots illustrate the association between irisin levels and various laboratory markers. Lower irisin levels were linked to dyslipidemia and hypertension. (c) Additionally, irisin levels were associated with hepatic ballooning scores.

Statistical differences between groups were assessed using the Mann–Whitney U or the Kruskal-Wallis tests. Correlations between variables were evaluated using Spearman's rank correlation coefficient (Spearman's ρ). ALT: Alanine aminotransferase; BMI: Body mass index; MASH: Metabolic dysfunction-associated steatohepatitis; T2DM: Type 2 diabetes mellitus.

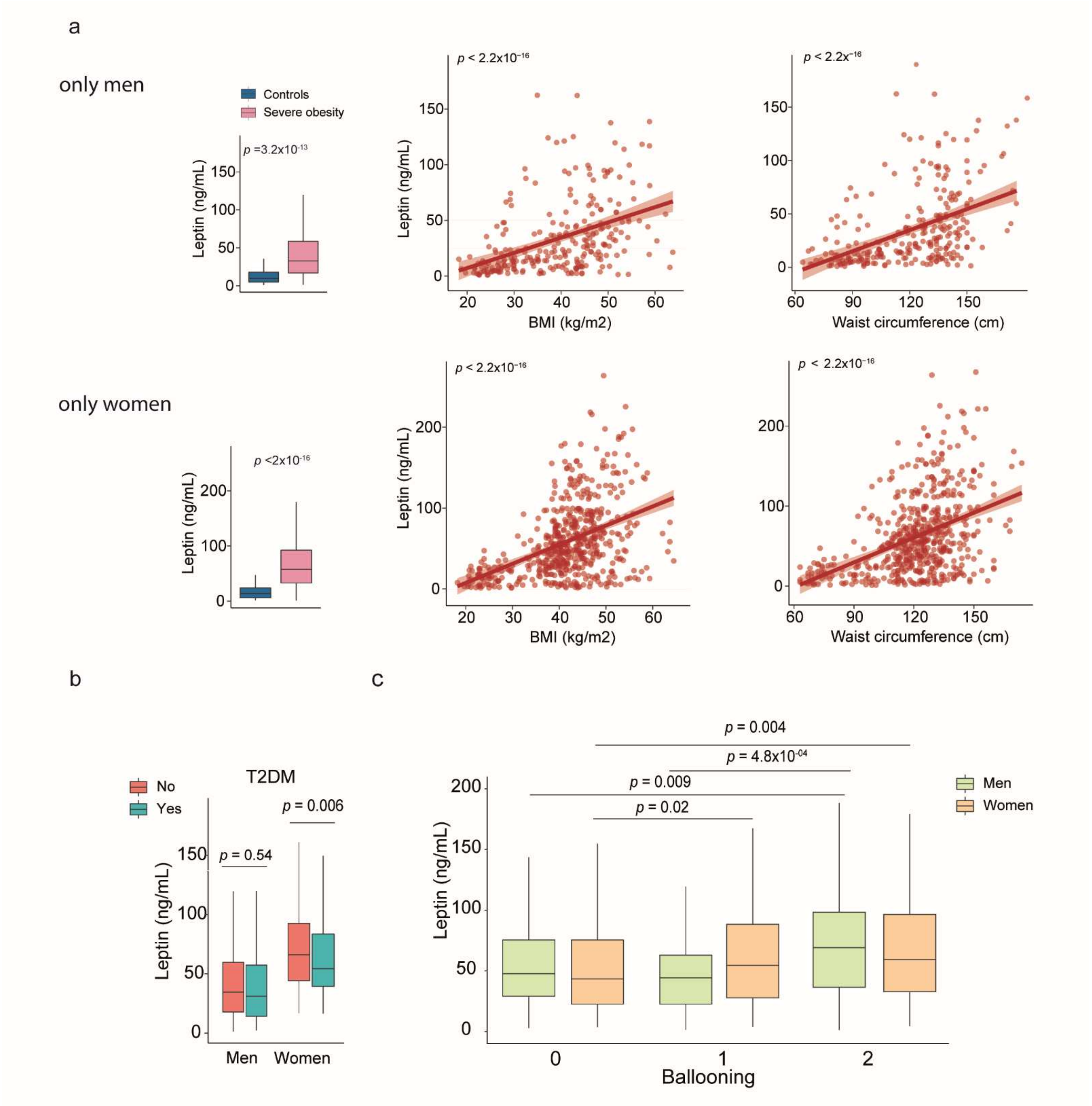

Supplementary Fig. S6. Leptin levels in men and women. (a) In cases of severe obesity, circulating leptin levels were elevated in both men and women, with no significant differences in their relationship to body size. (b) Likewise, the connection between leptin levels and ballooning scores was consistent across the sexes. However, the association with diabetes was observed only in women, attributed to higher leptin levels (refer to Figure 3 for values in the whole cohort).

Statistical differences between groups were assessed using the Mann–Whitney U test. Correlations between variables were evaluated using Spearman's rank correlation coefficient (Spearman's ρ). BMI: Body mass index; T2DM: Type 2 diabetes mellitus.

Supplementary Table S4. The effect of sex on the associations between liver histologic features and plasma leptin and adiponectin levels.

	Estimate	*P* value
NAS histological scoring system		
Leptin	0.0020	0.130
Women	0.0020	0.340
Men	0.0009	0.710
Adiponectin	-0.0400	0.004
Women	-0.0500	0.007
Men	-0.0500	0.090
Steatosis score		
Leptin	0.0004	0.740
Women	0.0004	0.800
Men	0.0006	0.800
Adiponectin	-0.0800	$1.3x10^{-6}$
Women	-0.0700	$3.7x10^{-4}$
Men	-0.1200	$1.2x10^{-4}$
Lobular inflammation score		
Leptin	-0.0010	0.490
Women	0.0001	0.990
Men	-0.003	0.250
Adiponectin	-0.0500	0.005
Women	-0.0400	0.040
Men	-0.0600	0.040
Ballooning score		
Leptin	0.0050	0.001
Women	0.0050	0.005
Men	0.0040	0.070
Adiponectin	0.0200	0.260
Women	0.0100	0.600
Men	0.0200	0.470
Fibrosis score		
Leptin	-0.0010	0.510
Women	-0.0010	0.650
Men	-0.0010	0.720
Adiponectin	-0.0020	0.920
Women	0.0001	0.990
Men	-0.0100	0.630

NAFLD: non-alcoholic fatty liver disease. The column "estimate" represents the estimated coefficients (β) for each predictor in the model. These values indicate the effect of each independent variable on the probability that the ordinal dependent variable takes higher values on its scale. If $\beta > 0$, increasing the predictor increases the probability that the response falls into a higher category. If $\beta < 0$, increasing the predictor decreases the probability of being in a higher category. If $\beta \approx 0$, the predictor has little to no effect. The results are presented together or separately based on sex.

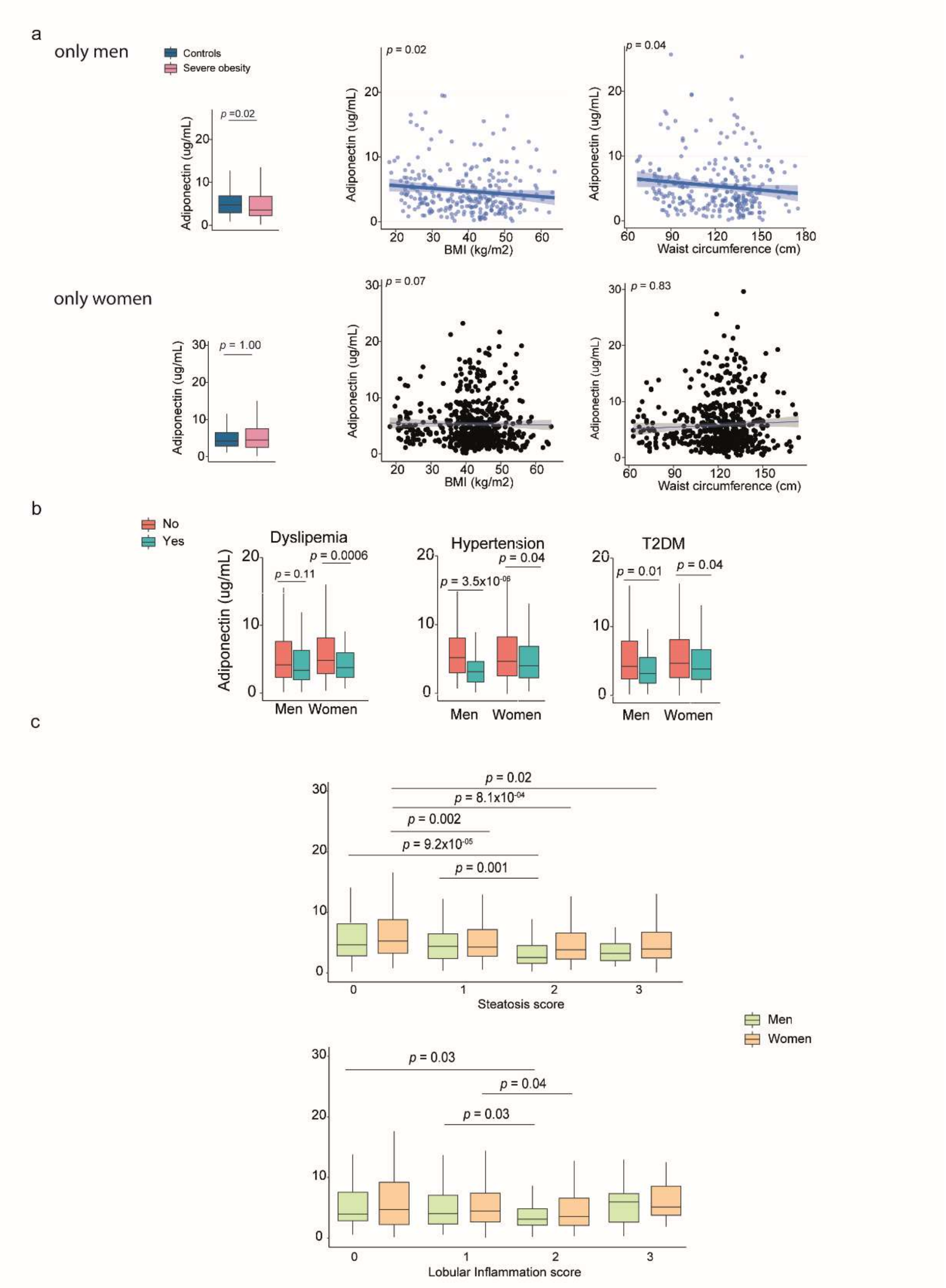

Supplementary Fig. S7. Adiponectin levels in men and women show notable differences: (a) Lower plasma adiponectin levels and their association with body size were found to be significant only in men. (b) The relationships between metabolic dysregulation and adiponectin levels were less evident in both men and women. (c) The connection between adiponectin levels and scores for steatosis and inflammation was consistent across both sexes. Statistical differences between groups were assessed using the Mann–Whitney U or the Kruskal-Wallis tests. Correlations between variables were evaluated using Spearman's rank correlation coefficient (Spearman's ρ). BMI: Body mass index; T2DM: Type 2 diabetes mellitus.
